# Considering future qualification for regulatory science in the early development of microphysiological systems: a case study of microthrombosis in a Vessel-on-Chip

**DOI:** 10.3389/ftox.2024.1513002

**Published:** 2024-12-06

**Authors:** Huub J. Weener, Heleen H. T. Middelkamp, Andries D. Van der Meer

**Affiliations:** ^1^ Bioengineering Technologies, University of Twente, Enschede, Netherlands; ^2^ Institute for Human Organ and Disease Model Technologies (hDMT), Eindhoven, Netherlands

**Keywords:** regulatory science, qualification, context of use, microphysiological system, organ on chip, endothelial cells, thrombosis

## Abstract

Microphysiological systems (MPS) and Organs-on-Chips (OoCs) hold significant potential for replicating complex human biological processes *in vitro*. However, their widespread adoption by industry and regulatory bodies depends on effective qualification to demonstrate that these models are fit for purpose. Many models developed in academia are not initially designed with qualification in mind, which limits their future implementation in end-user settings. Here, we explore to which extent aspects of qualification can already be performed during early development stages of MPS and OoCs. Through a case study of our blood-perfused Vessel-on-Chip model, we emphasize key elements such as defining a clear context-of-use, establishing relevant readouts, ensuring model robustness, and addressing inherent limitations. By considering qualification early in development, researchers can streamline the progression of MPS and OoCs, facilitating their adoption in biomedical, pharmaceutical, and toxicological research. In addition, all *in vitro* methods must be independent of animal-derived materials to be considered fully fit for purpose. Ultimately, early qualification efforts can enhance the availability, reliability, and regulatory as well as ethical acceptance of these emerging New Approach Methodologies.

## Introduction

With the rise and development of microphysiological systems (MPS) and Organs-on-Chips (OoCs) (Ingber, 2022), end-users and regulators are increasingly interested in these complex *in vitro* models as potential New Approach Methodologies (NAMs) for toxicology, safety pharmacology, and efficacy testing (Homan, 2023; Stresser et al., 2024). While there is a growing number of commercial OoC products on the market (Zhang and Radisic, 2017), the majority of reported OoC models are still at a low technology readiness level, and are mostly developed and used in an academic context. Developers of OoC models typically do not consider standardization and regulation early in their design process, hindering their possible future development into products that are qualified for use in commercial and regulatory contexts (Leung et al., 2022; Piergiovanni et al., 2024). For both acceptance by end-users and regulatory bodies, any *in vitro* model needs to go through a process of qualification that demonstrates that the model is fit for purpose.

**FIGURE 1:**
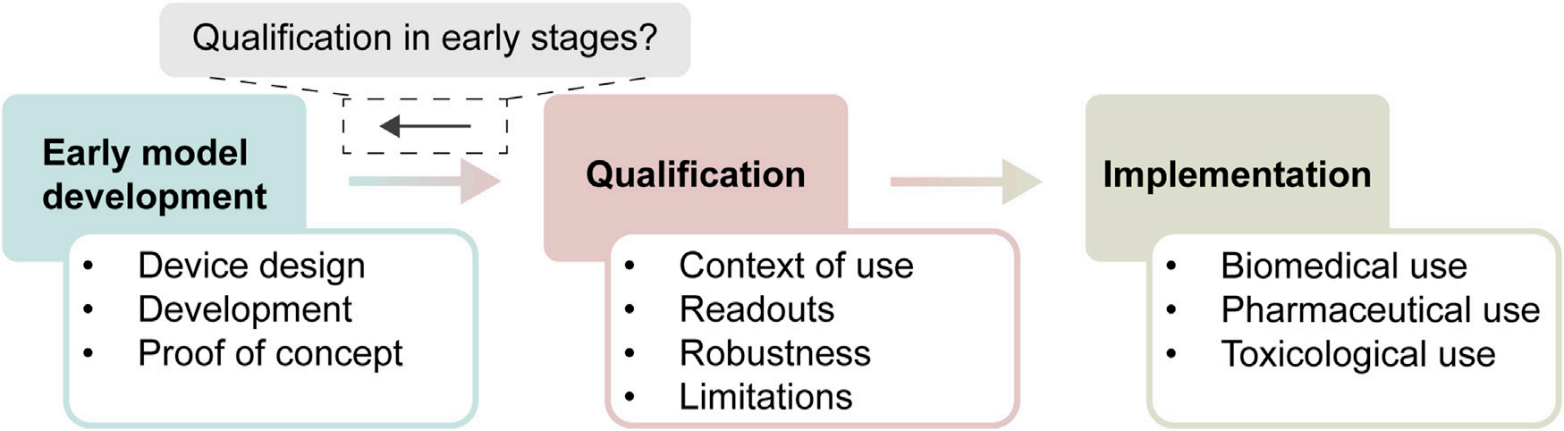
Timeline of development of new approach methodologies. In early development, models are designed, extensively characterized up to proof-of-concept, which is used to start the qualification process. Qualification contains different aspects that have to be answered, before the model can be deemed fit-for-purpose within its context of use. Subsequently, a qualified model is implemented for its context of use, where it sees standardized use within biomedical, pharmaceutical, or toxicological settings. The central question that we try to answer is to which extent aspects of qualification can already be considered during early stages of model development.

Qualification can be defined as a process that results in the “…conclusion that the results of an assessment using the model or assay can be relied on to have a specific interpretation and application in product development and regulatory decision-making.”(FDA, 2017). This qualification process includes not only the intended application of the model, but also how the model works, what its readouts tell us, and how variable and reproducible it is (Figure 1). Without qualification, the end-user cannot be sure that the obtained data from a model actually is fit to answer their question of interest. Stakeholders in the field of OoC identified qualification as an area of high importance for training of early developers of OoC models (Moruzzi et al., 2023). However, most qualification steps are typically taken when models near market implementation or widespread use within a company, and not during early model development. Most OoCs developed in an academic setting are not designed with qualification in mind, causing a large gap between the development and qualification of models (Ekert et al., 2020).

Why is qualification often overlooked in academic settings? One aspect may be that most early model developers focus on biotechnological innovation, using new fabrication methods, read-outs or complex tissue interactions. Most of this development is not primarily driven by a clearly defined intended use of the final model. A second aspect may be related to cost, with systematic studies of reproducibility and rigorous benchmarking of a new OoC model requiring resources that are not available in early research settings. A third aspect may be that early developers lack the knowledge and training in the key concepts and methods that are used in qualification. The central question is whether it is possible for early MPS and OoC model developers to consider future qualification, or whether this is fundamentally incompatible with their approach, setting or resources. In this manuscript, we address this question by systematically mapping key aspects of qualification on the work performed over the past years on development of a blood-perfused Vessel-on-Chip (VoC) model. We address the key points that regulatory instances provide as advice to NAM developers, and critically discuss the opportunities and limitations of making qualification an integral part of early MPS and OoC model Development.

### Blood-perfused Vessel-on-Chip

**FIGURE 2:**
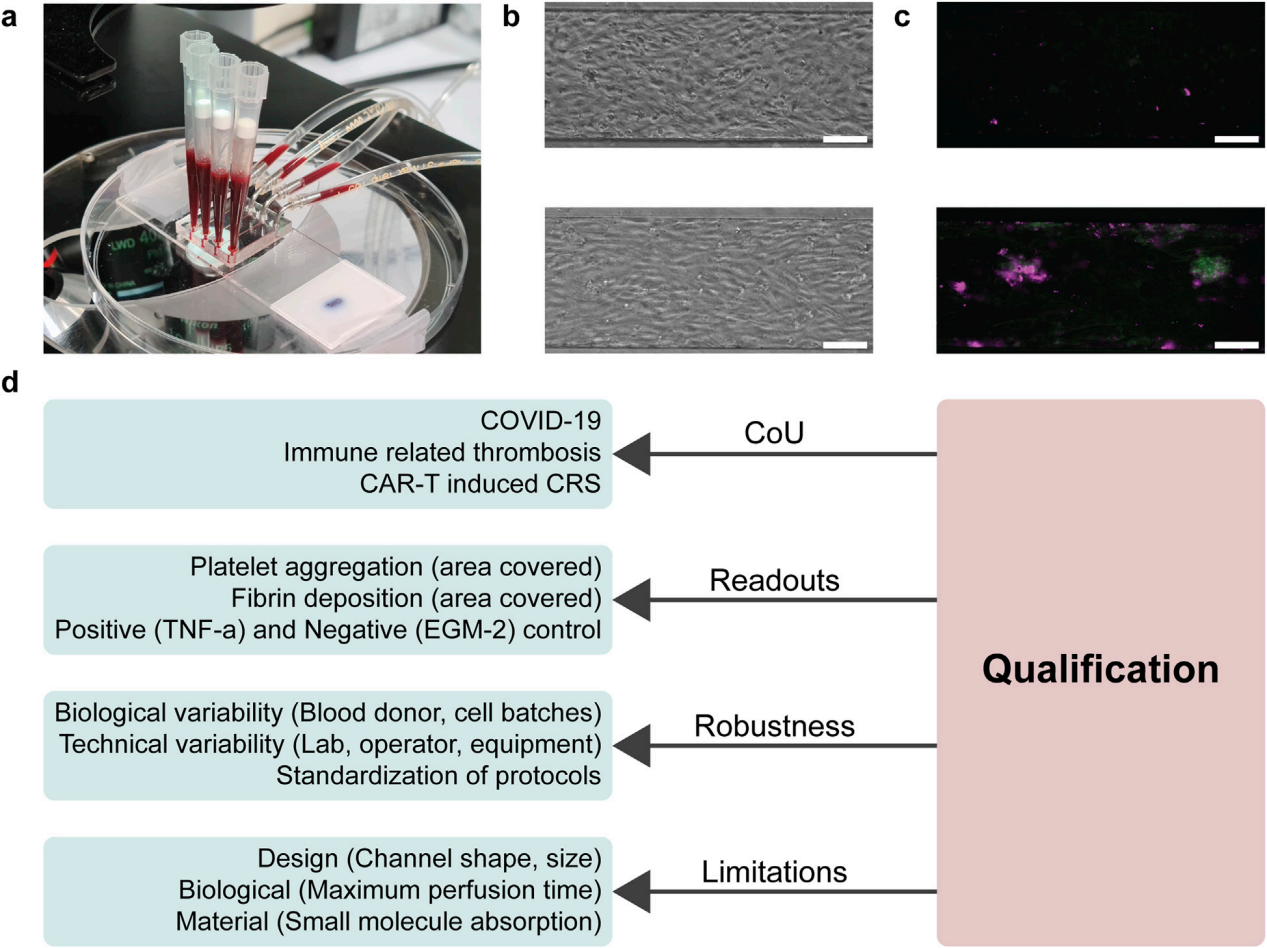
Overview of addressing aspects of qualification during early model development within the blood-perfused Vessel-on-Chip. **(A)** Photograph of the blood-perfused Vessel-on-Chip system, where blood is drawn by a syringe pump through the channels to create a physiological blood flow. **(B)** Typical morphological images of the endothelial monolayer in negative (top; EGM-2) and positive (bottom; 10 ng/mL TNF-α) conditions. Positive conditions show more elongated endothelial cells parallel to the flow direction. Scale bar, 100 µm. **(C)** Typical fluorescence microscopy images of platelet aggregation in negative and positive conditions. Positive conditions have elevated deposition of platelets (green) and fibrin (magenta). Scale bar, 100 µm. **(D)** Schematic of aspects of qualification that could be answered during early model development of the blood-perfused Vessel-on-Chip. For all criteria of qualification, certain aspects were found to already be addressed, mostly implicitly, in early development.

The blood-perfused VoC model has been applied in multiple research projects and its fabrication and associated protocols have been described in detail in previous publications (Jain et al., 2016; Albers et al., 2019). Briefly, it is based on a polydimethylsiloxane (PDMS) microfluidic device that contains four rectangular channels (14 mm × 300 μm × 50 μm; length × width × height), bonded to a PDMS-coated microscope slide (Figure 2A). Different endothelial cell sources, like Human umbilical vein endothelial cells (HUVECs) or Human induced pluripotent stem cell derived endothelial cells (hiPSC-ECs; LUMCi001-A) (Halaidych et al., 2018) are seeded at high density (15×10^6^ cells/ml) in two steps, first on the top half and then on the bottom half, forming a monolayer that covers all sides of the channels overnight (Figure 2B) (Albers et al., 2019). Thereafter, the endothelium can be treated with different stimuli, including those that mimic various disease states. With this approach, it is also possible to titrate concentrations of different medications, such as monoclonal antibodies against cytokines, to induce specific effects on the endothelium.

After treatment of the endothelium, the VoC is perfused with recalcified human whole blood, to simulate the acute thrombotic reaction that occurs after endothelial activation. This reaction is visualized and quantified using fluorescently labelled anti-CD41 antibodies and fibrinogen. After the channels are washed and fixated, the channels are imaged using a fluorescence microscope to calculate the area covered by thrombi within the channels (Figure 2C).

### Qualification framework

There are many available resources from regulatory bodies, governmental organizations and research institutes that outline the overall approach to qualification of NAMs, including MPS and OoC models (European Medicines Agency, 2016; FDA, 2020; Piergiovanni et al., 2021; 2024; Baran et al., 2022; Center for Drug Evaluation and Research, 2024; European Commission, 2024). Overall, the key aspects of a model that need to be defined and supported by data are the context-of-use, readouts, robustness, and limitations. All these aspects will be discussed in more detail in the respective sections below, and are summarized in Figure 2D. We will also critically consider the work on early development and application of the VoC model in the context of these aspects.

### Qualification of the blood-perfused Vessel-on-Chip model

#### Context of use

The context-of-use (CoU) of a NAM is defined by the FDA as the manner of application and purpose of an assay, including its characterizing conditions and parameters (FDA, 2020). It is important that early on in development, developers already identify the potential future CoU of their models: what exact mechanism of action or key event in toxicology or pharmacology can the model capture, and what functional requirements are needed for their model to be used as an assay for such events?

A well-defined CoU explains to future users what the MPS or OoC model does, and what uses it might have for their questions. A clearly defined CoU also enables future end-users to identify the existing models that may be fit for answering their particular questions of interest, and even allows side-by-side evaluation to identify the model that best fits their requirements (Homan, 2023).

Development of our VoC model started with no clearly defined CoU; instead, we were mostly interested in evaluating whether it was possible to develop an MPS that could capture aspects of thrombosis *in vitro*. Proof-of-concept studies were performed in which the VoC was treated with the inflammatory cytokine tumor necrosis factor (TNF)-α, and patterns of platelet adhesion upon human whole blood perfusion were analysed (Jain et al., 2016). Based on this proof-of-concept, we engaged in collaborative studies to apply the model in more defined CoUs, e.g., in tracking thrombosis as an unwanted side-effect of immunosuppressive monoclonal antibody drug candidates (Barrile et al., 2018), or novel therapeutic chimeric antigen receptor (CAR-)T cells. Moreover, *in vitro* modelling of microthrombosis in COVID-19 was explored by treating the VoC with patient plasma, with foreseen future use as a model for developing therapeutic strategies to counter this key pathophysiological process. This broad variety of applications also highlights the typical pragmatic and explorative nature of early academic research with MPS models.

Overall, the VoC does not have a well-defined CoU as required in a framework of qualification. Naturally, the VoC has a broad, and mostly implicit, CoU in that it i) captures key aspects of microthrombosis (platelet aggregation and fibrin formation), ii) does so on activated vascular endothelium, iii) also performs in an acute (hours to days) setting. Only when collaborating with other partners did we more sharply define respective CoUs on top of the broad foundational description. For example, in studying microthrombosis as a side-effect in CAR-T immunotherapy, we defined the CoU as capturing a ‘Key Event’ of disseminated intravascular coagulation in the context of cytokine release syndrome. This ‘Key Event’ was derived from an immune-related adverse outcome pathway (irAOP), describing the causal chain of events based on scientific evidence and a consortium of experts. Similarly, when using the VoC to study microthrombosis after treatment with patient plasma, the CoU was defined as microthrombosis due to endothelial activation in response to systemic inflammation in COVID-19.

#### Readouts

Each CoU requires a well-defined readout or endpoint that aligns with the models intended purpose. According to the FDA-NIH Biomarker working group, an endpoint is “a precisely defined variable intended to reflect an outcome of interest” (FDA-NIH Biomarker Working Group, 2021). For qualification of models, developers can choose between continuous readouts or endpoint measurements, but each must be relevant to the *in vivo* phenomenon being modelled. Regulatory bodies require that the connection between the readout and the mechanism of action is clearly defined and supported by evidence, as this determines the model’s CoU and capabilities (European Commission, 2024).

To support model claims, regulatory bodies require results obtained from positive and negative controls, as well as reference compounds that demonstrate a direct link between the model and the *in vivo* situation. A reference compound is seen as a previously developed drug used in humans, with well-characterized effects on the relevant tissues (Leite et al., 2021). While positive and negative controls routinely verify the overall technical effectiveness of a model’s readouts, reference compounds are adaptable and may vary depending on the specific CoU.

In our case study of the VoC model, an endpoint analysis was defined based on the assumption that the model could capture microthrombosis. We identified platelet aggregation and fibrin clot formation as key parameters and used fluorescence microscopy as the readout method for both. The model uses an anti-CD41 flow cytometry antibody to stain platelets and fluorescently labelled fibrinogen to stain fibrin deposits. Both labels are introduced 10 min before blood perfusion, enabling real-time tracking of platelet aggregation via fluorescence microscopy, or the measurement of final clot area after flushing excess blood (Manz et al., 2020). Positive and negative controls were based on earlier *in vitro* models, with TNF-α inducing the thrombotic cascade of endothelial cells, and EGM-2 culture medium serving as the negative control (Levi et al., 2006).

However, these controls only demonstrate the model’s ability to recapitulate microthrombosis under certain conditions and may not be applicable for other stimuli. For example, if a CoU involves microthrombosis induced by a monoclonal immune therapy, a reference compound with a similar mechanism of action is preferred. As an example, monoclonal antibodies against CD154, known to cause thromboembolic events in halted clinical trials (Sidiropoulos and Boumpas, 2004), were tested in the VoC model at relevant concentrations and successfully induced thrombosis (Barrile et al., 2018). This initial proof-of-concept of detecting a relevant thrombotic side-effect should be reinforced by testing other biologicals at drug-relevant concentrations.

#### Robustness

The robustness of a readout and endpoint must be evaluated within each CoU. Robustness is typically demonstrated through analyses of variability and reproducibility, encompassing both intra-test variability and inter-operator or inter-laboratory variability. While some variability is expected, the qualification framework requires that it remains within predictable ranges, based on identified influencing factors, to ensure reproducibility. To assess robustness, standardization is critical, often demonstrated through Standard Operating Procedures (SOPs), quality control of model components, and dynamic range criteria for positive and negative controls.

Intra-test variability can arise from factors such as the (biological) materials used. Since MPS and OoCs are more biologically complex than standard *in vitro* models, they may exhibit greater variability, which must be understood and managed. Each cell type used in the model must be thoroughly characterized to ensure it accurately replicates the intended pathological or toxicological effects. Additionally, both commercial and in-house cell batches often show some degree of heterogeneity in cell type or phenotype (Mertz et al., 2018). Commercially available cells are typically standardized and pre-characterized, while in-house produced cells must follow Good Manufacturing Practices (GMP) and established standards, such as those outlined by the International Society for Stem Cell Research (ISSCR) (Coecke et al., 2005; Pamies et al., 2022; Ludwig et al., 2023). These practices ensure high-quality cell sources, which can be further characterized for specific uses in the model. Regardless of the source, all cells should be assessed for variability within the developed model.

In our VoC model, we tested different types of endothelial cell sources to test the robustness of the model, showing similar patterns of increased platelet and fibrin deposition if treated with 10 ng/mL TNF-α. Furthermore, each new source of endothelial cells or freshly differentiated batches of hiPSC-ECs are tested using the positive and negative controls, to ensure their susceptibility towards the used stimuli. Moreover, we observed variability in platelet aggregation levels among blood donors, affecting both negative controls (pre-treatment with EGM-2 medium) and positive controls (pre-treatment with 10 ng/mL TNF-α). Due to donor service regulations, we cannot access information such as age or gender, limiting our ability to predict donor-related variability. To manage this, we established clear thresholds for platelet aggregation: <2% culture area covered for negative controls and >4% coverage for positive controls before including experimental data from a particular blood donor. Finally, we are drafting an SOP to standardize the fabrication and readout process of the VoC model, enabling other researchers to apply the model.

#### Limitations

Every choice made during the development of a system introduces limitations inherent to its design. These do not render the product flawed, but they do affect how data is interpreted and how experiments are designed for specific CoUs. Such limitations can arise from different aspects of the model, including constraints related to designs, materials, biological cells and tissues, or culture parameters.

Our VoC, lined with endothelial cells, focuses on the interaction between blood and endothelial cells. This differs from other flow-based systems might target platelet adhesion and fibrin deposition on collagen-coated surfaces, primarily addressing platelet activation in response to different treatments (Gandhi et al., 2024). Additionally, some systems incorporate multiple cell types, while our model is limited to endothelial cells, meaning it cannot account for tissue-resident immune cells (Gray and Farber, 2022) or organ-organ interactions (Jain et al., 2018). Thus, a comparison made with associated devices can assist in understanding how your developed model fits its CoU and therefore your system’s limitations. These limitations are inherent to the model and cannot be easily overcome.

Other design limitations relate to the dimensions and shape of the channels within the model. Different CoUs may require alternative channel sizes or the inclusion of hydrogels, which would necessitate significant redesigns. Furthermore, our VoC faces biological limitations, such as only being able to assess acute thrombotic effects. The maximum perfusion time which is currently achievable is 30 minutes. This prevents the study of long-term blood perfusion, drug pharmacokinetics, or repeated dosing scenarios.

Material-based limitations are also significant, particularly the absorption of small hydrophobic molecules by PDMS. This absorption has been extensively characterized and can limit the model’s accuracy in studies involving such molecules (van Meer et al., 2017). When materials other than PDMS are used, factors like oxygen permeability and cell adhesion properties may also impact cell behaviour (Sønstevold et al., 2023). Researchers should carefully consider material-specific properties of their model and be aware how this might limit normal cell behaviour in their model.

## Discussion

This case study shows that it is possible to incorporate qualification within early model development of MPS and OoC models, and highlights its importance. Certain qualification aspects, particularly related to readouts and model robustness, are already integrated in early development of our VoC model. This is typical for MPS models in early development towards generating proof of concept, as this often includes basic characterization. It would be useful to make the supporting data of these aspects of readouts and reproducibility explicitly available to end-users and other developers, for example, by uploading them in open repositories. Currently, early model developers might publish their gained results and characterizations split up over different publications, creating a fractured view of the characterization of the developed model. Developers should also be aware that characterization of robustness and readouts in their early model development, will not be enough to drive full qualification of their model; more data will typically be needed to demonstrate that the model is fit for a particular purpose.

Another aspect of qualification, namely, the limitations of the developed MPS or OoC, is typically something of which early model developers are often well aware. For example, one of the biggest disadvantages of using biological components is their natural variability, which is well-known in the field of cell culture. Particularly, the use of animal-derived products is widely recognized as a source of variability in MPS and OoC, including the model discussed here. Transitioning toward fully defined media compositions will be important for reducing batch-to-batch variability and enhancing the human relevance of *in vitro* models (Weber et al., 2024). For the endothelial cell cultures in our model, commonly used supplements like heparin, which is animal derived, can be replaced with biochemically synthesized alternatives (Douaisi et al., 2024). Similarly, animal-derived antibodies in our workflow can be substituted, as demonstrated by the use of chemical cell tracker DiOC_6_ to stain platelets within blood, achieving comparable readouts to antibody-based approaches (Westein et al., 2012). In addition, multiple other animal-derived components in our study, like rat tail collagen, would need to be replaced to obtain a full animal-free model. The claim of a robust system is only possible when all animal derived components are replaced, but standardization and optimization of these animal free methods is needed. As long as MPS and OoCs continue to rely on animal-derived products, they cannot be considered fully fit for purpose, limiting their adoption towards reliable NAMs. Still, it is highly recommended if we as a field want to develop MPS and OoC that contribute to developing robust, reproducible, and ethically sustainable models.

While defining a clear CoU is one of the most important aspects of future qualification of assays based on the model, it is also the aspect that early model developers typically tend to overlook. When models are first developed, developers tend to demonstrate broad or generic functions while more specific CoUs are typically established later on through dialogue with stakeholders who wish to adapt the model for their research needs. This transition from a focus on early proof of concept of a model towards using it in defined CoUs is not a given, as it requires multi-stakeholder collaboration. Researchers that do not have access to interaction with future users may focus repeatedly on innovation up to the point of proof of concept without defining a specific CoU that will be essential for future qualification. We advocate for both early model developers and end users to proactively look for collaborations, as we have noticed that it strongly facilitates the definition of a meaningful CoU.

Multiple stakeholders, particularly from the pharmaceutical industry, are advocating for increased qualification of academic models. The growing demand for qualified MPS and OoC models is driven by initiatives such as the IQ MPS Consortium (Tomlinson et al., 2024), which focuses on integrating these systems into industry workflows. These consortia not only promote MPS and OoC adoption but also generate lists of reference compounds relevant to various organ systems (Ainslie et al., 2019; Baudy et al., 2020; Hardwick et al., 2020; Peters et al., 2020; Phillips et al., 2020; Pointon et al., 2021). Early model developers have growing demands for these reference lists and can use them to benchmark their models against existing *in vivo* data, whether from animal models or human subjects (Stresser et al., 2024). These initiatives highlight the strong awareness in the field that building multi-stakeholder interactions will be essential to facilitate qualification of MPS and OoC, as well as their implementation as potential NAMs in regulatory science.

We argue that early model developers should implement our recommendations for addressing aspects of future qualification early in their workflow, as it will streamline subsequent stages of qualification and will facilitate dialogue with future end-users. Combined with later-stage improvements, like standardization and cost-efficient manufacturing, this approach could make MPS and OoC models widely accessible for end-users (Mastrangeli et al., 2019). For the field as a whole, an early focus on qualification means that developed MPS or OoC models will transition more often or more quickly into the phase of commercial assay development. Overall, qualification is essential for accelerating the implementation of MPS and OoC systems and should therefore be a key consideration from the outset of animal-free model development.

## Data Availability

The original contributions presented in the study are included in the article/supplementary material, further inquiries can be directed to the corresponding author.
